# Spirulina—An Invaluable Source of Macro- and Micronutrients with Broad Biological Activity and Application Potential

**DOI:** 10.3390/molecules29225387

**Published:** 2024-11-15

**Authors:** Izabela Podgórska-Kryszczuk

**Affiliations:** Department of Analysis and Food Quality Assessment, University of Life Sciences in Lublin, Skromna 8, 20-704 Lublin, Poland; izabela.podgorska-kryszczuk@up.lublin.pl

**Keywords:** *Arthrospira*, spirulina, microalgae, food fortification, vegan food, future food, bioactive compounds, sustainable food production

## Abstract

With population growth expected in the near future and the planet’s limited resources, alternative food sources are already being looked for. In this context, spirulina is called the food of the future due to its rich nutritional composition. This blue–green alga is primarily a valuable source of protein (55–70%) containing all essential amino acids. In its composition, it also contains unsaturated fatty acids, minerals, vitamins, and pigments, including the valuable protein–pigment complex—phycocyanin. Due to its high content of complete protein and minerals such as iron and calcium, it is an excellent addition to diets, especially those of vegans and vegetarians. Despite several limitations to the use of spirulina, including its distinctive marine flavour, low consumer awareness, or relatively high price, scientists are attempting to enrich many food products with the microalga. This is supported not only by the improved nutritional composition of the fortified product but also by spirulina’s impact on sustainable food production. Therefore, this review aims to create consumer attention by presenting spirulina as a valuable and sustainable food source with health-promoting potential and great future significance.

## 1. Introduction

Microalgae, due to their rich composition and valuable properties, are an excellent alternative food source and are described as the future of the food industry [1]. These organisms show several advantages compared to higher plants; mainly, these are (1) the lack of need to occupy limited land areas; (2) the possibility of cultivation in agriculturally unsuitable areas using non-potable or saline water; (3) shorter growth periods; (4) higher efficiency in the production of protein and bioactive compounds; and (5) the ability to control the production of beneficial compounds by regulating growth conditions [2,3,4]. Among microalgae, spirulina is the most well-known and widespread. The two most popular species are *Arthrospira platensis* and *Arthrospira maxima*. Spirulina is widely used in the production of food and dietary supplements due to its “generally recognised as safe” (GRAS) status given by the US Food and Drug Administration (FDA) [5,6]. Commercially, spirulina can be found in various forms, mainly dry powder, tablets, and capsules. Its recommended daily dose ranges from 0.25 g to 5 g [7]. Due to its unique composition, the World Health Organisation (WHO) has named spirulina a “superfood”. This microalga is an extremely rich source of protein, containing up to 70% of dry weight and all essential amino acids. It also contains essential fatty acids, numerous vitamins, minerals, and pigments, including chlorophylls and carotenoids [8]. Notable is that spirulina’s nutrients are highly bioavailable, making them rapidly absorbed by the body [7]. Noteworthy are the water-soluble and pigmented proteins extracted from spirulina, termed phycobiliproteins. Among them, phycocyanin has high production potential and offers many applications in the food, cosmetic, and pharmaceutical industries [9]. The valuable composition of spirulina makes it a popular choice for consumers who value a healthy lifestyle, as well as those who are particularly prone to deficiencies, including vegans and vegetarians [10].

Due to the predicted population growth in the upcoming years, ways are already being sought to provide food for the future. Increased demand for protein is leading scientists to intensify research on spirulina, a valuable source of this ingredient. In addition, numerous studies confirming the alga’s positive effects on the body and demonstrating its multifaceted importance in treating many diseases contribute to the growing interest in spirulina by consumers and the food and pharmaceutical industries [5]. Among other things, spirulina shows antioxidant, anti-inflammatory, and immune-modulating effects, supports the treatment of cardiovascular diseases, lowers blood pressure and glucose levels in diabetics, supports weight loss, and prevents neurodegenerative diseases [6,11,12]. Intensive research on bioactive compounds from microalgae also proves their anticancer effects [2]. Many scientific papers show spirulina’s potential in food fortification, aimed primarily at increasing protein or mineral content. Spirulina supplementation has been proven to have a positive effect on many staple foods, including bread [13], pasta [14], biscuits [15], yoghurt [16], kefir [17], sauce [18], and even meat products [19]. However, the large-scale implementation of microalgae faces several challenges, including low awareness of its nutritional benefits, less consumer acceptance of the enriched product of spirulina’s characteristic taste and smell, and its relatively high price. Already, scientists are focusing on solving these problems by taking various steps, such as promoting nutritional knowledge, improving microalgae production methods, and modelling its growing conditions [5].

The review presented here discusses the role of spirulina’s rich composition in determining its valuable properties. It presents methods of spirulina cultivation, demonstrates the microalga’s important function in food fortification, describes its effects on vegan and vegetarian diets, and provides possible therapeutic applications. In addition, issues often trotted marginally or overlooked in numerous works, i.e., the challenges of spirulina use, its impact on sustainable food production, and future research trends, are discussed. This review aims to look broadly at spirulina, a valuable source of many compounds with multidirectional biological activity and broad application possibilities.

## 2. Materials and Methods

Current knowledge of spirulina as an invaluable source of macro- and micronutrients with broad biological activity and application potential was analysed. The analyses focused mainly on the nutritional aspect of spirulina use. The microalga’s rich composition was described, affecting its valuable nutritional and therapeutic properties. Spirulina cultivation systems were characterised as a critical step in modelling the microalga’s production of specific compounds. The focus is on its use in food fortification and its significant role in sustainable food production. Special attention was paid to spirulina as an excellent supplement to vegan and vegetarian diets. Limitations in the use of spirulina and ways to overcome them are described. Prospects for further research are also presented.

The literature review includes 147 scientific publications, the majority from the last five years, 2019–2023, and the current year (more than 84%). It cites one piece of legislation and an article on the International Agency for Research on Cancer website. Publications were searched using interdisciplinary and specialised scientific databases: PubMed, ScienceDirect, Scopus, Web of Science, Google Scholar, Springer, Taylor & Francis, and Wiley Online Library. The following keywords were used to search for relevant publications: *Arthrospira*, spirulina, microalgae, phycocyanin, bioactive compounds, proteins, amino acids, food fortification, vegan food, future food, and sustainable food.

## 3. Results

### 3.1. Spirulina Composition

Spirulina’s composition can significantly vary depending on nutrient availability and culture conditions (light intensity, salinity, pH) [9,20]. Undeniably, spirulina is a source of high-quality protein (55–70%), carbohydrates (15–25%), dietary fibre (8–10%), fats (6–9%) and minerals (7–13%). It contains numerous vitamins, pigments (chlorophylls, carotenoids, and phycocyanin), and phenolic compounds [21,22,23,24].

#### 3.1.1. Protein

Proteins are essential for the proper functioning of the body, play an important role in various physiological processes, and contribute significantly to the nutritional quality of the diet. The structure of proteins determines their digestibility, bioavailability, and the physiological reactions they release in the organism. Protein bioavailability refers to how a protein can be absorbed, utilised, and incorporated into the body’s various metabolic functions. Protein bioavailability and amino acid composition are significant because they determine the nutritional quality of the protein source [25]. However, it has recently been proposed to expand the concept of protein quality to include its environmental impact, including the amount of fertiliser and pesticides used, carbon footprint, water footprint, land footprint, or biodiversity footprint [26]. In this context, it seems spirulina is an excellent source of protein.

Microalgae, including spirulina, are identified as an alternative source of protein for malnourished populations [8]. Spirulina contains significantly higher amounts of protein (55–70%) compared to common foods (Table 1) considered valuable sources of protein, including beef (17.4–22%), chicken (19–22%), fish (19.20–22%) or soybeans (35.35–40.30%) [8,22,27].

In addition, it is important that spirulina proteins are highly digestible (85–95%) [22] due to the structure of the microalga’s cell wall consisting mainly of proteins, carbohydrates, and lipids [29]. This contrasts with the plant cell wall, which consists of cellulose indigestible to humans [22]. Proteins are formed from essential amino acids, which must be supplied to the body because they cannot be produced independently, and non-essential amino acids, which are synthesised in the body [30]. Spirulina is an extremely valuable source of protein, as it contains moderately high amounts of essential and non-essential amino acids (Table 2). The amino acid profile is influenced by culture conditions (including the culture medium used), so it may vary from one research work to another [31].

Spirulina contains all the essential amino acids for the human body, including lysine, leucine, histidine, phenylalanine, methionine, threonine, tryptophan, and valine [23]. It is also highly digestible (higher than plant products) [35], as shown in Table 3.

Spirulina protein is key in preventing protein-energy malnutrition. It has been shown to have promising techno-functional properties, including emulsifying activity, foaming properties, good solubility, and retaining water and oil [36,37]. These properties are desirable for food manufacturers, particularly those looking to address the needs created by the growing interest in vegan and vegetarian diets [37]. Spirulina protein has been shown to exhibit similar adsorption kinetics and surface tension reduction as whey protein isolate, one of the most popular stabilisers. In contrast to typical animal proteins, microalgae proteins can stabilise emulsions and foams over a wide range of pH and ionic strength. This is related to the characteristically low isoelectric point (pI = 3–4) [38]. The proper ability to retain water and oil in food matrices, as demonstrated by spirulina protein, plays an important role in the texture and structure of a food product [36].

**Figure 1:**
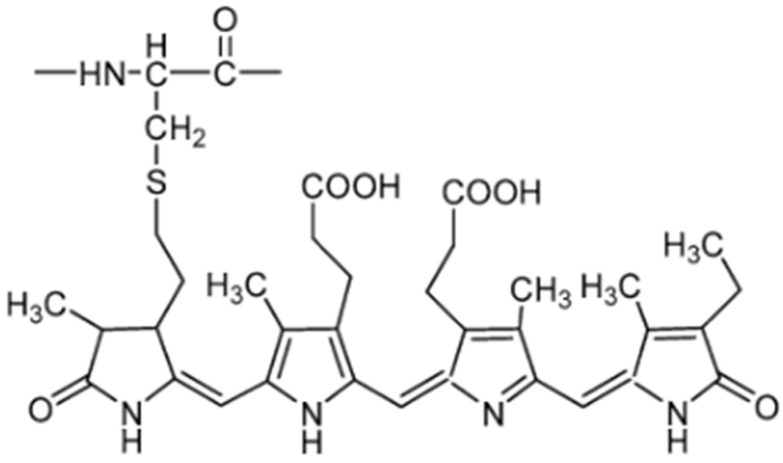
Chemical structure of phycocyanin from *A. platensis* [7].

The protein fraction of spirulina contains up to 20% phycobiliproteins. These are blue-coloured proteins that include both phycocyanin and allophycocyanin [9,39]. Phycocyanin (Figure 1) has many uses as a dye in the food and cosmetic industries. Being a food ingredient, this protein pigment can only be obtained by filtered water extraction of dried *A. platensis* biomass. In the cosmetics industry, phycocyanin has found use as an ingredient in lip balms, hair dyes, and anti-ageing creams. The pharmaceutical industry uses it as an ingredient in medicines and supplements. This protein pigment is a natural blue dye with many valuable properties, including antioxidant, anti-inflammatory, immune-modulating, and anticancer properties. The phycocyanin chromophore is the tetrapyrrole molecule phycocyanobilin B, and it is probably responsible for spirulina’s high antioxidant activity. The phycocyanin chromophore is more effective at inhibiting oxidation than natural antioxidants, such as zeaxanthin, alpha-tocopherol, and caffeic acid [9,40,41]. Due to its fluorescent properties, phycocyanin can also be used as a biofluorescent marker for staining DNA, WBCs (White Blood Cells), RBCs (Red Blood Cells), and platelets. The pigment has applications in fluorescence microscopy, flow cytometry, fluorescence immunoanalysis, and histochemistry. As a fluorescent probe, it can detect cyanobacterial blooms [41]. Phycocyanin has also shown antimicrobial activity against acne-causing *Propionibacterium acne* and *Staphylococcus epidermidis* [42]. It has been proven to inhibit the growth of antibiotic-resistant bacteria such as strains of *Klebsiella pneumoniae, Escherichia coli*, *Staphylococcus aureus*, and *Pseudomonas aeruginosa*. Phycocyanin isolated from spirulina also has antifungal activity against pathogens such as *Alternaria alternata*, *Aspergillus niger*, *Fusarium oxysporum*, *Fusarium graminearum*, and *Fusarium culmorum* [43]. Due to phycocyanin’s unique properties and widespread use, numerous attempts are being made to improve methods for its extraction and increase production efficiency by exposing *A. platensis* to abiotic stresses, such as exposure to temperature and light or changing the culture medium [41].

#### 3.1.2. Carbohydrates

Microalgae synthesise carbohydrates through photosynthesis and carbon fixation. They play various roles, including structural components of cell walls or metabolic energy stores. They consist mainly of sugars such as glucose (97%), rhamnose (2.7%), and mannose (0.4%) [43]. It is noteworthy that by manipulating the parameters of spirulina cultivation, the carbohydrate content can be significantly increased from 15–25% to as much as 50–70%. Factors affecting carbohydrate content include nutrient availability (mainly nitrogen and phosphorus), salinity, temperature, and light intensity. In the study, Markou et al. [44] showed that by limiting phosphorus, A. platensis can produce increased amounts of 1.3:1.6-β-glucans (between 20 and 34% of dry biomass). Due to the bioactive functions of β-glucans (antioxidant, immunomodulatory, anticancer, and antimicrobial activities), they are of great interest as functional ingredients for food production. Unlike fungi, yeast, or grains, microalgae are not widely used as producers of β-glucans, but the growing global market for these ingredients is prompting the search for new sources. In a recent study, it was shown that spirulina using even wastewater from the anaerobic fermentation of the water hyacinth macrophyte (*Eichhornia crassipes*) as a culture medium produced significant amounts of β-glucan (4–18% of dry weight) [45]. In addition, the rich composition of spirulina’s oligosaccharides means that its consumption supports intestinal microflora growth. The acidic polysaccharides present in A. platensis, thanks to their content of sulphate esters, sulphate groups, and amino residues, are capable of inducing the synthesis of tumour necrosis factor, and polysaccharide extracts from the alga show chemoprotective activity [23]. It has been proven that the heteropolysaccharide (SP90–1) present in spirulina, consisting of glucose, rhamnose, galactose, glucuronic acid, xylose, and fructose, exhibits immunostimulatory and anticancer effects by inhibiting the growth of A549 lung cancer cells [43].

#### 3.1.3. Lipids

The fat content of spirulina ranges from about 6% to 9% [24], and its lipid profile consists of saturated fatty acids (SFA), monounsaturated fatty acids (MUFA), and polyunsaturated fatty acids (PUFA) [23]. Many unsaturated fatty acids are essential for the human body, and among them, omega-6: arachidonic acid (AA), gamma-linolenic acid (GLA), and omega-3: docosahexaenoic acid (DHA) and eicosapentaenoic acid (EPA) are contained in spirulina [11,24]. It is crucial to supply the body with an adequate amount of lipids, as they are components of cells and ensure the proper functioning of many metabolic reactions [4]. GLA from spirulina may help regulate blood pressure or lower cholesterol levels, which can be used to treat cardiovascular disease. It has also been shown to have antioxidant properties that help delay the effects of skin ageing [24]. EPA and DHA positively affect the cardiovascular and nervous systems and play an essential role in foetal development [46].

#### 3.1.4. Minerals, Vitamins, Pigments, and Phenolic Compounds

Spirulina is a food with numerous minerals and vitamins (Table 4). It contains significant amounts of potassium, phosphorus, calcium, iron, sodium, and magnesium. It also includes zinc, copper, manganese, and selenium in its composition. The microalga is a source of vitamins, mainly B vitamins (B1, B2, B3, B6, and B12) and provitamin A or vitamin E. It also contains biotin and folic acid in its composition. In addition, spirulina is rich in natural pigments, including phycocyanin (14–20%), as well as chlorophyll (1%) and carotenoids (0.5%) [47]. These three pigments differ not only in colour and composition but also in solubility, stability, and commercial importance [48]. Phycocyanin, in addition to its obvious colouring function, can be successfully used as a dietary supplement due to its functional benefits. It is commercially available as a liquid extract or in powder form [49]. Due to the demand for natural and health-promoting products, the global market for phycocyanin continues to grow. It is expected to rise to USD 245.5 million by 2027 and USD 279.6 million by 2030. It should be noted that the remaining spirulina biomass after pigment extraction remains a valuable source of vitamins, minerals, and antioxidants [40]. Spirulina contains phenolic compounds, by-products of metabolism, which in the alga perform protective functions against stress caused by such factors as ultraviolet radiation, pollution, or pathogens. The content of these compounds determines spirulina’s antioxidative effect [22]. The content of polyphenolic compounds in spirulina methanol extract is 7.8–44.5 mg GAE/g. Polyphenols that occur in the highest amounts in the microalga are benzophenone, propanediamine, phenylacridine, piperidine, carbanilic acid, pyrrolidine, oxazolidin-2-one, and dinitrobenzoate [43].

### 3.2. Spirulina Cultivation

**Figure 2:**
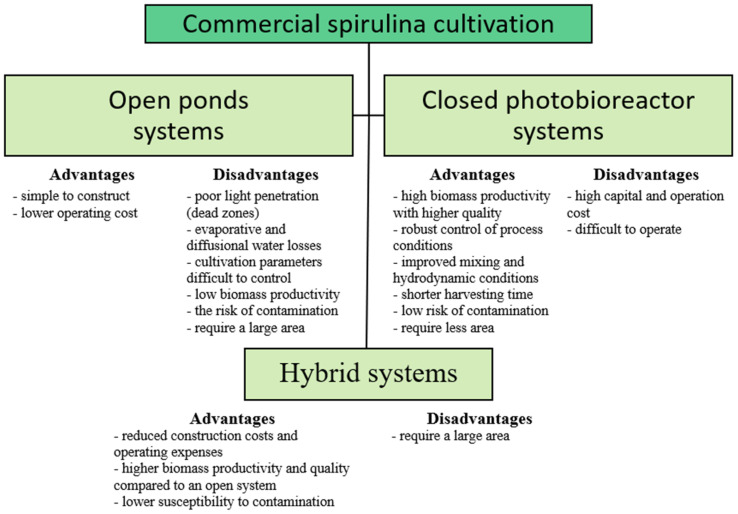
Comparison of spirulina production in open, closed, and hybrid systems [41,54].

Commercial spirulina production is well-developed worldwide, with most cultivation in tropical or semi-tropical regions due to the conditions favouring the microalga’s growth. In Europe, spirulina is the most widely cultivated microalgae, yielding nearly 150 tonnes of dry biomass annually [53]. Spirulina is primarily grown using tanks in two systems: open and closed. In open systems, spirulina culture is mostly in contact with the environment (except for parts of open reactors in controlled rooms or greenhouses). These systems are the most commonly used due to the cheaper cost of construction and operation. The big challenge in an open system is controlling the temperature of the culture medium, as much energy is required to heat large volumes of culture. In contrast, closed systems provide a physical barrier from the environment, making their control less demanding. This allows for higher biomass productivity, with higher quality, in less time [5]. Closed photobioreactor systems are characterised by improved mixing, hydrodynamic conditions, extended gas retention time, and thus high mass transfer. Hybrid systems combining closed photobioreactors with open systems are also used in spirulina cultivation. These are designed to reduce construction costs, operating expenses, pollution, and CO_2_ losses. A comparison of the three systems used in spirulina cultivation is shown in Figure 2.

Commercial cultivation of spirulina is carried out in many countries. The largest companies involved are Cyanotech Corporation (Kailua-Kona, HI, USA), Earthrise Nutritionals LLC (Irvine, CA, USA), Parry Nutraceuticals (Chennai, India), Inner Mongolia Rejuve Biotech Co. Ltd. (Ordos, China), Yunnan Green A Biological Project Co., Ltd. (Lijiang, China), Boonsom (Chiang Mai, Thailand), IGV Biotech GmbH (Nuthetal, Germany), and Algae Biotechnologia (Sao Paulo, Brazil). For economic reasons, most companies use open channel ponds to grow spirulina. Biomass productivity in these systems is generally governed by their location, light intensity, pond depth, dissolved oxygen content, CO_2_ supply, mixing, or salinity. Spirulina’s high tolerance to harsh environmental conditions allows it to be grown in open systems, even with possible exposure to contaminants [54]. Among other factors contributing to the popularity of spirulina cultivation is the wide range of conditions it tolerates. Its adaptation to different environments is influenced by its physiological and metabolic plasticity, resulting from its high intra-species diversity, independent of phylogenetic affiliation or geographic location. Spirulina shows the most abundant growth in an alkaline environment (pH 9.5–11), which significantly reduces the risk of contamination of the culture with other photo-synthetic microorganisms. It prefers moderate to relatively high temperatures (25–35 °C), with a light intensity of 300–500 μmol photons m^−2^ s^−1^ [54,55]. Direct sunlight is not recommended for outdoor cultivation of spirulina due to possible photoinhibition. Up to 30% sunlight is recommended to avoid overheating the culture medium. For optimal spirulina biomass, stirring and aeration of the crop are also essential. It aims to distribute temperature, light, and CO_2_ evenly. The optimal mixing speed is in the range of 5–60 cm s^−1^. In modelling the production of specific compounds by spirulina, the composition of the culture medium is crucial. For example, nitrogen availability affects the accumulation of phycocyanin, as this protein pigment selectively degrades under nitrogen-deficient conditions [54]. The nitrogen source is also essential—higher productivity is obtained using urea than ammonium or nitrate [56]. In addition, suitable precursors such as succinic acid and monosodium glutamate increase the synthesis of phycocyanin [54]. The synthesis of carotenoids, chlorophyll, phycocyanin, and allophycocyanin can be increased using a modified blue–green 11 medium [56]. Due to its optimal composition, the standard substrate for spirulina cultivation is the Zarrouka substrate, which provides good biomass production. However, this medium’s limitation is its economic issue. Other commonly used culture media include Rao, OFERR, CFTRI, and George’s [54].

### 3.3. Spirulina in Food Fortification

Most ongoing research on food enrichment with spirulina biomass primarily aims to improve the product’s nutritional profile. This research remains mainly on a laboratory scale, and relatively few spirulina products are currently available on store shelves [9,57]. The online sales include protein sezam bar with spirulina (Go Raw, Northbrook, IL, USA), mint protein chocolate, almond orange bar and berry chocolate with spirulina (Nutrezy, Mumbai, India), lemon spirulina bar (Lubs GMBH, Luebeck, Germany), muesli with spirulina (One Day More, Poznań, Poland), honey with spirulina (Pasieki Rodziny Sadowskich, Srebrna, Poland), fusilli pasta with spirulina (Pastificio Minardo, Modica, Italy), spirulina tagliatelle (Priméal, Peaugres, France), Spirulina blue water (FUL foods, Delf, Holand), basil spirulina salad dressing (McMaster’s Muskoka Fine Foods, Bracebridge, ON, Canada).

Numerous scientific studies have shown that spirulina has been added to many traditional foods, such as baked goods, pasta, cookies, and dairy products (Table 5). An undoubted benefit of using microalgae in food fortification is the product’s significant increase in protein content and antioxidant activity. Another important advantage of fortifying foods with spirulina, especially for people who exclude animal products from their diet, is increased mineral content, especially iron and calcium [18]. However, in scientific papers, only a few products undergo mineral analysis (Table 5), so this is not experimentally confirmed in every product. Interesting matrices to which spirulina has been added are fermented dairy products. It has been proven that microalgae fortification can improve the growth of probiotic bacteria [17,58]. The addition of spirulina to biscuits positively affects texture parameters, such as hardness and crispness, which are essential for this product type [15]. Due to its increased water-holding capacity, spirulina’s addition is associated with a change in texture, an improvement in viscosity, a reduction in syneresis, and even an increase in shelf life [9,59]. When designing foods fortified with spirulina biomass, a noticeable change is expected in the product’s colour and sensory properties. Therefore, selecting the appropriate amounts of the additive and considering the product matrix is important. In most of the studies conducted, spirulina is added at a relatively low level due to the characteristic aroma of the algae. The addition is usually a maximum of 1–2% in dairy products [16,58,60], while it is higher in grain products such as pasta and biscuits, reaching up to 10–20% [61,62]. Foods fortified with spirulina are expected to be intensely green. This makes foods of this particular colour a suitable matrix for microalgae enrichment, as demonstrated in studies of spirulina-enriched pesto. Adding microalgae at 1% contributed to the very high consumer rating of the green sauce, and the colour was better rated than in the basil control sauce [18]. However, depending on the other ingredients used and the heat treatment, the colour can turn brown, affecting consumer acceptability [9]. Phycocyanin, a protein pigment found in spirulina, is also a growth opportunity in the market for coloured beverages that do not contain synthetic dyes [63]. Synthetic dyes are added to many products to make them more attractive. The natural blue colour is rarely found in food, making phycocyanin highly innovative in the food industry [9]. By the FDA in the Code of Federal Regulations Title 21. 73.530 (21 CFR § 73.530), spirulina extract has been exempted from certification and approved as safe for colouring a wide range of products, including soft drinks and alcoholic beverages with less than 20% alcohol content, ice cream and frozen desserts, cottage cheese, chewing gums, candies, icings, toppings, yoghurts, puddings, custards, gelatin, breadcrumbs, cereals (except extruded cereals), condiment mixes (not heated), dips, and sauces [64]. The European Union requires clear labelling of products with spirulina extract, as it is classified as a dye without an E number [65]. Phycocyanin is soluble in water but, unfortunately, sensitive to high temperatures and pH changes [63]. However, this problem is relatively easy to solve, as demonstrated after baking crostini. Adding vegetable oils, such as extra-virgin olive oil or sunflower oil, rich in α-tocopherol, can protect the phycocyanin from degradation during heat processing [66]. In addition to its apparent effect on the colour of the product, the addition of spirulina can exhibit stabilising and emulsifying activity, as has been demonstrated in ice cream [67].

### 3.4. Spirulina in a Vegan and Vegetarian Diets

Diets that avoid the consumption of animals or even animal products are becoming increasingly popular in developed countries. Conversion to vegetarian or vegan diets can be for several reasons, including health, environmental, religious, cultural, or taste preferences [75]. Such a diet has been proven to positively affect the body, including the gut microbiota, lowering blood pressure, reducing weight, and preventing cardiovascular disease, type 2 diabetes, and cancer [76]. However, a plant-based diet, despite its positive effects on the body, if not carefully planned, can cause deficiencies in several nutrients that it does not provide or are poorly absorbed from the gut. These include high-quality protein, long-chain omega-3 fatty acids (EPA and DHA), iron, calcium, zinc, selenium, iodine, and vitamins B12 and D [77,78,79].

Protein, rich in essential amino acids, is key to the body’s growth, proper development, and metabolism [25]. Proteins of animal origin, derived from meat, fish, or dairy, are generally highly digestible (90–95%) [80], and the body absorbs much of their amino acids. Undoubtedly, plant-based proteins offer health and environmental benefits, but due to their lower digestibility and lower content of essential amino acids (especially leucine, lysine, or methionine), they have less anabolic effect than animal proteins [25,81]. Leucine (Leu), isoleucine, and vanillin are extremely important among the essential amino acids because of their role in neuronal function, blood glucose and insulin regulation, and protein metabolism [82]. Lysine (Lys), on the other hand, is an essential part of the binding site for some proteolytic enzymes, especially trypsin [25]. Methionine (Met) is required for methylation, a factor in preventing neural tube defects and osteoporosis, and is necessary for growth and ensures healthy skin and nails. This amino acid is also required for selenium and zinc absorption, T-cell proliferation, and differentiation [83]. The lower digestibility of plant proteins is due to their complex structure and anti-nutritional content [25]. Phytates, saponins, and tannins can affect protein absorption [30]. Therefore, plant proteins often require additional formulation to remove anti-nutrients, increase digestibility and nutrient density, and improve functional properties (e.g., water and oil retention capacity) [80]. Given this problem, spirulina appears to be an ideal source of protein for those on a vegan or vegetarian diet. This microalga contains 60–70% protein by dry weight, which is higher than in any other natural food. It contains all eight amino acids essential for humans and has a high digestibility range of 80–90% [50]. Spirulina contains high amounts of essential amino acids, often consumed in insufficient quantities by people on plant-based diets. For example, Liestianty et al. [21] showed that alga contains 55 mg/g Leu, 30 mg/g Liz, and 14 mg/g Met; in contrast, baby spinach (raw) has 1.81 mg/g Leu, 1.42 mg/g Liz, 0.28 mg/g Met; broccoli (tenderstem) cooked 1.86 mg/g Leu, 1.88 mg/g Liz, 0.48 mg/g Met; chard (ruby), raw 2.21 mg/g Leu, 1.98 mg/g Liz, 0.53 mg/g Met; rocket (raw) 1.93 mg/g Leu, 1.55 mg/g Liz, 0.25 mg/g Met. [84].

Another risk that a vegan and vegetarian diet may entail is a deficiency of the vitally important long-chain omega-3 fatty acids EPA and DHA [85]. Their primary sources are oily fish and seafood [86]. Long-chain omega-3 fatty acids are essential in maintaining health while performing several important immune, neurological, cardiovascular, and cognitive functions [30,83]. Low levels of EPA are associated with inflammatory imbalances, attention deficit disorders, arthritis, autoimmune diseases, eczema, and psoriasis. The consequences of DHA deficiency can include cognitive dysfunction, depression, aggression and impulsive violence, cardiovascular disease, and menopausal problems. DHA is necessary, especially during pregnancy, because it is their deficiency that can be linked to premature birth or postpartum depression. Maternal intake of DHA is important for the neurological development and function of the infant [83,85]. The precursor of EPA and DHA is also linolenic acid (ALA), but the ALA conversion rate into these two fatty acids is very low [30,83]. The human conversion rate of ALA to EPA and DHA is about 5–8% [86]. Spirulina is a good source of fatty acids, but most are omega-6 fatty acids [22], which are not lacking in a plant-based diet, as nuts, seeds, avocados, olive oil, or sunflower oil provide them [83]. Therefore, spirulina can supplement the diet of those on a vegan or vegetarian diet, but it does not entirely cover the need for long-chain omega-3 fatty acids.

Many plant-based sources of iron are available, including spinach, pumpkin seeds, almonds, cashews, chard, lentils, sesame, and tofu [83]. Although vegetarians generally consume as much iron, or even slightly more, than omnivores, they are at risk of iron deficiency. This is due to the lower bioavailability of nonheme iron from plant sources compared to heme iron from animal sources. Reduced absorption of nonheme iron is related to its ease of binding to inhibitors (e.g., fibre, polyphenols, and tannins) [30,83]. Maintaining appropriate iron levels is essential for protein synthesis, energy transport and storage, oxygen transport, and many other processes involving metabolic functions related to muscle activity, bone strength, growth, immunity, and the nervous system [30]. As a microalga with a high iron content (0.7 mg Fe/g) [87], spirulina can be a good source of iron in vegan and vegetarian diets. This is supported by the fact that most of the spirulina’s iron is bioavailable (0.45 mg Fe/g) [87]. For example, there is 0.15–0.25 mg Fe/g in cereals, considered a valuable source of iron. In addition, its absorption can be significantly inhibited due to iron-chelating phytates [88]. Iron from spirulina is also better absorbed and has no toxic effects compared to ferrous sulphate, which is often used in dietary supplements and causes diarrhoea [22]. In addition, as microalgae and cyanobacteria can take up metals from the substrate, research is ongoing to develop an iron biofortification process to enrich spirulina with this element. In a recent study by Kougia et al. [87], *A. platensis* was cultured with an increased concentration of iron in the culture medium, which did not interfere with the alga’s growth. This promising research direction requires further attention to select an appropriate source of iron to make it as bioavailable as possible to humans.

Since dairy products such as milk, yoghurt, and cheese are the main sources of calcium, people on a plant-based diet are at risk of deficiencies in this element. Calcium is essential for bone growth, and deficiencies can lead to low bone mineral density, which can cause osteoporosis in the future [89]. A recent meta-analysis involving 74 studies and 166,877 people found that vegans have significantly lower calcium intake than vegetarians and omnivores [90]. Unfortunately, the bioavailability of calcium in plant-based products is variable due to compounds inhibiting its absorption, such as oxalic acid and phytic acid [30]. Many green leafy vegetables, such as spinach, are rich in calcium but also contain oxalic acid, which reduces its bioavailability to about 5% [89]. Spirulina contains calcium and phosphorus in amounts comparable to milk [88], with high bioavailability. In addition, spirulina maintains the proper ratio of calcium to phosphorus. For this reason, there is no danger of decalcification, which can occur when the amount of phosphorus in food increases [21,22,88].

A study in the United Kingdom found that iodine and selenium intake was deficient among women following vegan and vegetarian diets [91]. Selenium is an essential trace element in the human diet, and its sources are primarily meat, fish, and grain products [92]. As a component of selenoproteins, this element plays an essential enzymatic and structural role, acts as an antioxidant and thyroid hormone catalyst, supports the proper functioning of the immune system, supports fertility, and reduces the risk of miscarriage. Selenium deficiency can cause mood deterioration and, in more serious cases, Keshan disease (cardiac muscle dysfunction) and kidney disorders [69,93]. It has been proven that spirulina-enriched foods can be rich in selenium [69], but attention should be paid to the bioavailability of this element during supplementation. In rat studies, selenium from spirulina was less bioavailable than selenium from sodium selenite and selenomethionine. However, the study’s authors stressed that it is still biologically useful but is metabolised differently due to its form [92].

Spirulina can also be an important source of iodine in vegan and vegetarian diets. This group is particularly vulnerable to a deficiency of this element because the primary source of this element is eggs, fish, or dairy products. Table salt iodisation has not been mandated in all parts of the world, so it is significant for people who do not eat animal products to consume algae [94,95]. It is estimated that 35–45% of the world’s population is affected by iodine deficiency. This is associated with serious health consequences. People with mild deficiency are at risk of thyroid enlargement, benign thyroid nodules, and the development of endemic goitre. Severe iodine deficiency in adults manifests as hypothyroidism, goitre, mental disability, and reduced fertility. Children may develop goitre, deafness, impaired intellectual/physical development, and cretinism [95].

As mentioned, people on plant-based diets are also particularly vulnerable to vitamin B12 deficiency, as it is found almost exclusively in foods of animal origin [83,96]. This vitamin is an essential cofactor for the methylation processes involved in DNA and cell metabolism and for producing blood cells and nerve tissue [89]. Low levels of vitamin B12 can adversely affect memory, energy, mood, and nerve function [83], and persistent deficiency leads to serious conditions, including megaloblastic anaemia and peripheral neuropathy [89]. Unfortunately, spirulina contains an inactive analogue of vitamin B12, which cannot be absorbed in the human gut. Cyanobacteria can use pseudovitamin B12 as a cofactor, but it shows no physiological activity in mammals and is not considered a reliable source for humans [96].

### 3.5. Spirulina in Sustainable Food Production

The world’s population is growing steadily, and it is estimated that by 2050, it will reach 9.7 billion [97]. It is already estimated that about 1 in 9 people worldwide is malnourished, and the most essential factor is protein-energy malnutrition, that is, lack of an adequate supply of calories and protein [4]. To meet the demand for increased food production, the number of livestock is expected to increase significantly [98]. However, animal husbandry and feed production use nearly 80% of all agricultural land and contribute only 18% of global caloric production and 37% of total protein production [99]. Livestock production contributes significantly to climate change through high greenhouse gas emissions and gradual deforestation worldwide associated with feed crops [98]. Nearly 10 million hectares of deforestation are covered annually, and the expansion of agriculture is responsible for nearly 90% of it (50% for plantations and 39% for cattle grazing) [99]. Modern livestock production accounts for about 18% of global greenhouse gas emissions. This comprises ruminant enteric fermentation, methane release (rebound), and manure management [100]. Cattle farming is recognised as a significant contributor to global warming [98]. Rising temperatures are causing heat waves, droughts, erratic rainfall, floods, and other extreme incidents [99]. Crops are expected to be significantly affected by climate change [4]. This, in turn, leads to reduced production, which poses a direct threat to food security [99]. As a result, food futurologists are predicting significant changes in the type of food consumed. New products will partially replace some of today’s most popular ingredients [3]. In addition, changing consumer preferences and growing nutritional awareness will drive the development of food products to improve health and reduce the risk of lifestyle diseases [101].

Microalgae, including spirulina, are a promising alternative strategy for reducing the environmental impact of human development while increasing the value of end products [9]. Spirulina has excellent potential and several attractive features for large-scale sustainable food production. These are primarily high biomass yields per unit area and the ability to grow on wasteland using non-potable water and even saltwater [4]. Cyanotech Corporation (Kailua-Kona, HI, USA) has launched Hawaiian Spirulina^®^, which is produced using seawater. This is supported by the fact that spirulina production using seawater reduces the need for water (seawater accounts for 97.5% of all water on the planet) and reduces the risk of contamination [5]. This is extremely important given that agriculture is responsible for 72% of freshwater intake globally, mainly for irrigation [99]. The high salt content of seawater limits its use in agricultural systems but does not eliminate the possibility of using it in microalgae cultivation. Using seawater to produce spirulina biomass is a low-cost, sustainable approach that, as research has shown, is also highly effective. Seawater is rich in sodium, potassium, chloride, calcium, and magnesium, and it also contains other compounds needed for microalgae metabolism, such as iron, phosphorus, and nitrates [102]. An experiment by Villaró et al. [103] proved that although the protein content of *A. platensis* biomass produced using seawater was lower, the content of essential amino acids (leucine, isoleucine, and valine) was higher. The researchers also showed stimulation of carotenoid synthesis, mainly zeaxanthin and lutein, and a significant increase in oleic and eicosenoic acid content. It has been confirmed that the composition of spirulina biomass produced using seawater is suitable for food applications [103].

Another advantage of cultivating spirulina is that it requires much less land than conventional agriculture, such as poultry or vegetable farming, about 49 to 132 times [104]. Spirulina offers more nutrients per acre than any other food available. The alga’s rapid growth means its proteins require 20 times less land than soybeans, 40 times less than corn, and 200 times less than meat cattle. Moreover, spirulina proteins use 1/3 the water compared to soybeans, 1/5 compared to corn, and only 1/50 the water needed for beef protein [3]. Microalgae have a higher photosynthetic capacity compared to land plants. Their ability to capture CO_2_ can contribute to reducing the carbon footprint in the atmosphere [105]. As a result, even a gradual, incremental intake of spirulina as an alternative source of dietary protein could free up lands presently used for growing forage or grazing. The freed-up land could be reused by reforestation [100].

The microalgae biomass includes many components, the amount of which can be regulated by modifying culture conditions. Spirulina is mainly used as a source of protein, which it produces when all nutrients are available. However, changes in the medium’s components can alter the alga’s biomass composition, converting proteins into energy-storage compounds such as carbohydrates and lipids [105]. For example, in the study by Bezerra et al. [102], significantly higher carbohydrate content and biomass productivity than a control culture using Zarrouk medium had spirulina cultured with seawater. The factors driving the increase in carbohydrate content and, thus, the reduction in protein in spirulina biomass are the medium’s salinity and the reduction in the nitrogen and phosphorus source. To maintain the medium’s osmotic balance, the microalgae direct metabolism towards producing and accumulating low-molecular-weight osmoregulating carbohydrates such as sucrose and trehalose [102]. Unfortunately, one of the main disadvantages of growing spirulina is the high cost of the commonly used culture medium. The cost of a litre of Zarrouk substrate, mainly used by many companies to date, is approximately USD 0.08 [106], about 35% of the total cost of producing algal biomass. However, in addition to using seawater as a culture medium, as mentioned above, intensive research is underway to look for alternative sources of nutrients to grow spirulina to minimise its growing cost [104]. It has been suggested, for example, cassava flour, taro flour, sweet potato flour, table sugar [107], and urea [108]. Attempts have also been made to use wastewater as a medium for spirulina biomass production. This is a highly ecological and economical solution, as wastewater often contains many nutrients, such as nitrogen and phosphorus, which can be absorbed by the microalgae and converted into biomass through photosynthesis [109]. For example, wastewater from palm oil mills [109], distillery wastewater [110], olive oil pressing wastewater [111], and paper mill wastewater [112] have been used for this purpose. The use of wastewater for algae cultivation requires its proper treatment because, in addition to nutrients, it also contains contaminants that can lead to the collapse of the culture [109]. Spirulina’s unique ability to assimilate chemical oxygen demand and nutrients from selected wastewater is key in promoting closed-loop economy applications. In addition, due to its ability to bind carbon and nitrogen and adsorb metal ions, spirulina is attracting interest in various sectors dealing with environmental issues, aiming to achieve carbon neutrality and develop renewable energy sources [9].

### 3.6. Therapeutic Uses of Spirulina

Due to the abundance of health-promoting compounds found in spirulina, it is widely used in the production of dietary supplements. These supplements are available in various forms—the most popular form is powder, but capsules, tablets, flakes, frozen spirulina, and phycocyanin extract are also available [113]. A study by Rutar et al. [113] found that dietary supplements with spirulina (*n* = 46) are a good source of calcium, phosphorus, potassium, and selenium and contain essential and non-essential amino acids and polyunsaturated fatty acids. Although they contain large amounts of iron, their form (Fe^3+^) is less bioavailable. By making the microalga an effective accumulator of trace elements, there is concern about the accumulation of toxic elements (cadmium, mercury, lead, arsenic). However, the mentioned studies have shown that spirulina supplements do not significantly contribute to the intake of toxic trace elements and do not pose health risks. A survey by Janda-Milczarek et al. [114] showed that the mineral content of spirulina supplements (*n* = 33) is influenced by both the form of the supplement and the method of growing the microalga. The authors proved that powdered supplements showed significantly higher potassium, magnesium, and iron content while having significantly lower sodium content. Supplements made from organically grown microalgae were a better source of iron, while those derived from conventionally grown had higher phosphorus, calcium, and strontium contents. Due to its GRAS status and clinically proven lack of health risks, spirulina supplements are widely used. In addition, the demand for natural products makes consumers more likely to reach for them to support health than pharmaceuticals [11].

Several preclinical and clinical studies have demonstrated the promising therapeutic effects of spirulina. It has been proven to exhibit anti-inflammatory and anti-hypertensive properties and improve metabolic parameters. Supplementation causes weight loss and helps control cholesterol levels. In addition, the microalga may positively affect conditions such as type 2 diabetes, hypertriglyceridemia, and some autoimmune diseases. Numerous studies further suggest potential benefits for bone health, protection against liver damage, or reducing or inhibiting the growth of cancer cells [11,115,116]. Miczke et al. showed that in overweight patients with hypertension, three months of regular spirulina consumption improved BMI and weight and resulted in improved blood pressure. Miczke et al. [117] showed that in overweight patients with hypertension, three months of regular spirulina consumption improved BMI and weight and resulted in improved blood pressure. A meta-analysis by Moradi et al. [118] found that spirulina supplementation significantly reduces body weight, body fat percentage, and waist circumference, especially in obese individuals. Ghaem Far et al. [119] confirmed that spirulina significantly reduces systolic blood pressure, diastolic blood pressure, serum triglycerides, total cholesterol, and low-density lipoprotein levels. Kariz et al. [120] showed that spirulina supplementation as an adjunct therapy to metformin produces reasonable long-term glycaemic control and control of glycaemic levels in people with type 2 diabetes. In addition, spirulina has a beneficial effect on atherosclerotic receptors without any side effects. Spirulina’s hepatoprotective effects may be related to enhancing antioxidant defence mechanisms, antiproliferative effects, and inhibiting inflammatory cytokines/mediators. This was demonstrated in the Mohamed study [121] on rats, where spirulina prevented liver damage. In a survey by Moradi et al. [122], spirulina supplementation significantly reduced sleep disturbances and stress levels, which in the patients studied increased quality of life. Spirulina, thanks to its antioxidant and anti-inflammatory effects, also has neuroprotective significance, exerts a positive effect on glial cell activation, and aids in the treatment of neurodegenerative diseases, including Alzheimer’s disease, Parkinson’s disease, and multiple sclerosis [6].

Although spirulina is a promising complementary therapy for many conditions, further clinical studies are needed to confirm its efficacy over a more extended period of use in a diverse population in terms of ethnicity, lifestyle, or gender. The mechanisms of action of the microalga also need to be thoroughly elucidated, and the optimal supplement doses for supporting the treatment of specific conditions and the duration of treatment should be determined [11,115]. Developing new, effective forms for spirulina use, including nanotechnology products, is also a challenge to address [43].

### 3.7. Limitations of Spirulina Use

Although the FDA has given spirulina GRAS status, its use has several limitations. Similarly, the number of food products containing microalgae is growing yearly, but in Western cultures, spirulina products are not yet a common food component found on store shelves [57]. Consumers’ acceptance of spirulina may have difficulties due to its intense fishy taste and smell [123]. This is because even a small amount of strong odorant can impact the smell of a quality product [124]. The complex aroma of spirulina consists of numerous volatile organic compounds (VOCs), including hydrocarbons (mainly branched hydrocarbons, at more than 37% of total VOCs), aldehydes, ketones, nitrogen-containing compounds, esters, and furans, among others. In the study by Moran et al. [125] of the numerous branched hydrocarbons found in *A. platensis*, the researchers detected such compounds as heptane, 3-ethyl-2-methyl-; heptane, 2,4-dimethyl-; octane, 4-methyl-; octane, 5-ethyl-2-methyl-. Of the aromatic hydrocarbons, the significant compounds were benzene, methyl-, and benzene, 1,3-bis(1,1-dimethylethyl)-. Spirulina was also characterised by a significant amount of the acyclic aldehyde propanal, 2-methyl-, and the representative of cyclic aldehydes was β-cyclocitral. Saturated aldehydes are generally attributed with aromatic notes similar to earth and hay, while unsaturated aldehydes can impart oily and greasy odours [126]. Of the diketones in spirulina, 3,5-heptadien-2-one, 6-methyl- was detected. Diketones are characterised by an odour reminiscent of seafood. The primary odorant among cyclic ketones was β-ionone. This compound, along with β-cyclocitral, is derived from the enzymatic degradation of β-carotene. From the group of acyclic ketones, the researchers detected significant amounts of 2-propanone, which is responsible for the green fragrance notes. Also, an important compound of spirulina was 2-heptanone, 6-methyl-, which was included in the camphor family of fragrances [125]. In general, acyclic ketones are associated with desirable aromas in foods. Saturated ketones are compounds with sweet, floral, and fruity notes, while unsaturated ketones are associated with green aroma notes [127]. The researchers also found significant other compounds, such as 1-hexanol and 1-octen-3-ol. Some unsaturated alcohols, such as 1-octen-3-ol, can significantly affect odour. It is described as earthy, grassy, mushroomy, and oily [125]. Of the other volatile compounds in *A. platensis*, furan, 2-pentyl-, and trichloromethane have also been described. Furans are attributed to undesirable aromas of oils and fats imparting notes of bean, grassy liquorice, and tobacco odours [127].

There are several ways to solve the problem of microalgae odour. These include avoiding synthesising molecules with unpleasant aromas, avoiding their release through encapsulation, masking with other aromas, and removing them at the processing stage. Growth conditions, environmental factors (light intensity, temperature, pH), and nutrient availability have significantly impacted the production of volatile and odorous compounds in microalgae [124]. Therefore, by adequately modelling growing conditions, biomass with different sensory properties can be obtained that could be used to formulate food and feed products [128]. Due to its intense colour and aroma, the addition of spirulina to food products is very low and limited to a maximum of a few percent [16,60,72,129]. Microencapsulation is a promising strategy to account for the higher concentration of spirulina in an enriched product without affecting overall organoleptic properties. A study by da Silva et al. [62] showed that microencapsulation of *A. maxima* biomass by spray drying allowed as much as 20% addition of the alga to biscuits without losing sensory value. Microencapsulation of spirulina biomass has also been shown to preserve its antioxidant potential under adverse conditions (pH 1.2) and inhibit denaturation at elevated temperatures (100 °C) [130]. Encapsulation also weakens the green colour of the microalgae’s biomass [5]. Another proposed strategy to solve the problem of intense algal flavour is to mask the taste, smell, and/or colour by incorporating the biomass into dark or green food matrices, such as chocolate [33], pesto [18], or broccoli soup [131]. Another masking strategy can be the addition of algae to a savoury snack [124]. Deodorisation may also be an alternative for removing undesirable substances from algae-derived food products. Deodorisation is the final steam stripping process in the purification and refining of algae products, ensuring quality by removing volatile compounds, oxides, and free fatty acids (FFA) that can cause unpleasant aromas [124]. Another approach used to deodorise *A. platensis* biomass proposed by Cuellar-Bermúdez et al. [132] may be using chemical solvents (ethanol, acetone, and hexane). Researchers have shown that ethanol reduces marine odour and flavours from fatty acids, pigments, and proteins without affecting their sensory and nutritional properties.

Another limitation to spirulina use is consumers’ poor knowledge of the alga’s rich composition and potential health and environmental benefits. A recent Lucas et al. [133] survey found that about 50% of respondents (*n* = 442) did not know spirulina. A study by Kamenidou et al. [134] showed similar results on a larger group of respondents (*n* = 795), 57% unaware of spirulina. In another survey conducted by Lafarga et al. [57], as many as 85% of people (*n* = 3084) said that “there is a great lack of information about microalgae”. Of those surveyed, only 36.7% had tried microalgae or ingredients derived from them, and most had consumed them only once. In addition, respondents were asked to rate the main reasons limiting the consumption of microalgae. It was suggested that one reason could be the low number of microalgae introduced in commercial products, the lack of knowledge of where to buy them, and the lack of habit of consuming them. The intense green colour of the algae was also viewed negatively.

A critical factor in increasing consumer interest in consuming spirulina and products containing the alga is the realisation that such products are extremely valuable for their health and the environment. This was confirmed in a study by Lafarga et al. [57], in which consumers’ willingness to try and pay for spirulina increased after being provided with information about its healthfulness. Drawing attention to the favourable properties of spirulina improves consumer attitudes [135]. Weinrich and Elshiewy’s research [10] shows that consumers who prefer healthy lifestyles, vegetarians, and those who limit their meat intake are open to new foods. These consumers are also focused on organic production and are interested in the availability of information about food products. A study by Fantechi et al. [136] found that nutritional neophobia is a major obstacle to accepting spirulina-enriched foods. Iannuzzi et al. [135] studied preferences for spirulina and insect-based foods. The researchers showed that consumers reject products due to neophobia if they are made entirely of unfamiliar ingredients. In contrast, associating new ingredients (such as spirulina) with familiar foods reduces rejection. Grahl et al. [137] analysed three products with spirulina: pasta, vegan dried meat, and sushi. In their study, pasta was the most popular food, confirming that spirulina acceptance increases with familiarity with the product. In terms of consumer acceptability, foods containing spirulina have an advantage over other enriched foods, such as insects, because there is a greater chance that consumers will accept them. This is primarily because spirulina is perceived as a plant-based product [136].

An important factor in making spirulina more acceptable is its price. The cost of producing a kilogram of dried microalgae biomass is about USD 5, a high price compared to products such as wheat, rice, or corn, which typically cost less than 1 USD·kg^−1^. Lowering the production cost of algae can reduce its price. The production of spirulina od-beans is carried out in outdoor conditions using photobioreactors with open or closed systems. Higher productivity and biomass quality are achieved in closed photobioreactors, but this is associated with higher production costs. Therefore, open photobioreactors are the most widely implemented and are cheaper to build and operate. Another strategy currently being developed to reduce the cost of spirulina production is to modify the composition of the culture medium, looking for cheaper substitutes for particular ingredients or using alternative media, as described in the section above. Another issue is the development of new technologies for harvesting and drying microalgae, which could reduce costs [5,138].

### 3.8. Future Trends

Growing demand and the depletion of essential resources caused by population growth are increasing the market for protein ingredients, which are key in human nutrition. Due to environmental concerns, alternative protein sources are already being intensively searched for [139]. Increased consumer interest in health-promoting products and clean-label foods presents a promising future for microalgae as new food ingredients. In addition, microalgae-derived proteins’ functional properties (emulsifying, foaming, gelling) are prompting their use as substitutes for non-animal proteins [140]. Other industries, such as pharmaceuticals, nutraceuticals, and cosmetics, are also keen to use compounds derived from microalgae, as they are sustainable and renewable raw materials [41]. In this context, spirulina is important, so research on its use in human nutrition will be intensified, and the number of products with the alga will undoubtedly increase. However, further development of industrial production of spirulina with reduced costs and increased production capacity will be needed. It will also be necessary to develop new technologies to rapidly detect pathogens threatening spirulina crops and new, environmentally and consumer-friendly methods of controlling them [5].

Due to its superior protein content to other food crops, spirulina shows high potential for expressing therapeutic proteins. Also, in regulatory and food security matters, it appears to be unapologetically promising, as there is a negligible risk of genes escaping into the food chain due to spirulina’s asexual reproduction. However, no biological drug based on the algal platform has been introduced due to the lack of suitable genetic tools. In this regard, it is also necessary to develop new systems for growing spirulina to be suitable for producing biopharmaceuticals according to FDA CGMP (Current Good Manufacturing Practice) guidelines [141]. This prospect for the future will surely be the focus of many researchers.

A future approach will be the broader use of microalgae in nanotechnology, a rapidly growing multidisciplinary field. Interest in microalgae in this field is mainly due to their rapid biomass growth and easy cultivation method. Green synthesis of nanoparticles is an environmentally safe method and eliminates high energy inputs, and the resulting nanoparticles have favourable biocompatibility and non-toxic properties. Recent research highlights the potential of using spirulina to biosynthesise silver nanoparticles, which could find practical applications in many areas of biomedicine, pharmaceuticals, or food technology. Interest in synthesising silver nanoparticles is due to their diverse applications, such as food packaging and preservation, antimicrobial, anti-diabetic, anti-inflammatory, and anti-cancer effects, as well as their ability to treat water or use it as biological sensors [142,143]. The results of recent studies indicate the potent cytotoxicity of silver nanoparticles obtained by green synthesis using soluble polysaccharides isolated from spirulina against cancer cells [143]. This raises great therapeutic hopes for the future, but further research is needed to optimise the properties of the nanoparticles for their application in medicine.

Given spirulina’s rich composition, it is inevitable that the following research trend will be to identify new uses for the microalga and further research focusing on evaluating its health-promoting properties [5]. Already, intensive research is being carried out into spirulina’s anticancer effects, among other things [144,145]. Given the current therapeutic options and their side effects, searching for new solutions and compounds with anticancer activity is urgent [146]. This is a significant research direction because, according to the latest data from the International Agency for Research on Cancer, there were nearly 20 million cancer cases in 2022, and the incidence is expected to jump dramatically to as many as 35.3 million cases by 2050 [147]. In this context, further development of knowledge and new research techniques will be needed to efficiently extract, identify, and purify compounds with potential anticancer applications.

## 4. Conclusions

The review presents the latest data on spirulina, an invaluable source of macro- and micronutrients with a wide range of biological effects and applications. Microalgae is an excellent source of protein, which can prevent malnutrition in a population that is growing yearly. In addition, its cultivation is environmentally friendly—it does not need the already limited land areas, and it can use non-potable or seawater and even waste substrates, which has implications for sustainable food production. All of this is causing a recent increase in interest in spirulina, not only by conscious consumers but also by the food, cosmetic, and pharmaceutical industries. There are numerous attempts to incorporate spirulina biomass into many food products, and the alga itself is being sold as a dietary supplement. The growing number of studies aimed at filling gaps in existing knowledge is an opportunity to address currently problematic issues, including increasing awareness or maximising biomass production at the lowest possible cost. With new technologies and genetic tools, spirulina’s enormous potential will be fully realised.

## Figures and Tables

**Table 1:** Comparison of spirulina protein content to selected foods.

Product	Protein Content [%]	Reference
spirulina	55.00–70.00	[8,22]
beef	17.40–22.00	[8,27]
chicken	19.00–24.00	[8,27]
fish	19.20–22.00	[8,27]
parmesan cheese	36.00	[8,27]
skimmed milk powder	36.00–37.00	[8,27]
peanuts	25.80–26.00	[27,28]
wheat	11.88–13.20	[22,28]
rice	7.76–10.30	[22,28]
sunflower seeds	20.78	[28]
pumpkin seeds	30.23	[28]
soybeans	35.35–40.30	[8,22,27]

**Table 2:** Amino acid composition of spirulina.

Amino Acid Content [mg/g]			References
Essential	leucine	39.69–61.70	[21,32,33,34]
	tryptophane	8.50–10.00	[21,34]
	methionine	8.07–17.10	[21,32,33,34]
	phenylalanine	19.02–33.30	[21,32,33,34]
	lysine	22.62–34.00	[21,32,33,34]
	threonine	33.00–39.61	[21,32,33,34]
	isoleucine	25.34–36.40	[21,32,33,34]
	valine	27.89–45.00	[21,32,33,34]
	histidine	10.00–27.39	[21,32,33,34]
Non-essential	proline	22.92–27.00	[21,32,34]
	tyrosine	20.34–30.70	[21,32,33,34]
	glycine	21.26–34.30	[21,32,33,34]
	serine	20.35–33.00	[21,32,33]
	arginine	26.13–44.70	[21,32,33,34]
	alanine	42.80–50.20	[32,33,34]
	aspartic acid	57.9–63.10	[21,33,34]
	glutamic acid	83.9–92.00	[21,33,34]
	cysteine	4.60–7.00	[21,32,34]

**Table 3:** Digestibility of selected spirulina amino acids compared to amino acid digestibility of chickpeas and unshelled mung beans; adaptation from [35].

Amino Acids	Amino Acids Digestibility [%] *		
	Spirulina	Chickpeas	Unshelled Mung Beans
leucine	86.0 ± 3.1	68.5	62.3
isoleucine	84.2 ± 2.8	68.8	76.0
lysine	77.5 ± 9.5	44.4	56.5
methionine	84.1 ± 7.6	59.8	60.6
phenylalanine	95.3 ± 4.1	60.5	65.2
threonine	82.5 ± 2.6	53.8	43.6
valine	87.1 ± 5.0	64.1	64.5
proline	41.4 ± 5.7	33.2	32.6

* study performed on humans.

**Table 4:** Mineral and vitamin composition of spirulina; adaptation from [21,27,50,51,52].

Minerals [mg/100 g]		Vitamins [mg/100 g]	
Potassium	1400–1600	Thiamine (Vit. B1)	3.5–4.8
Phosphorus	800–1000	Riboflavin (Vit. B2)	4–5.5
Calcium	700–1500	Niacin (Vit. B3)	14–15
Sodium	250–900	Pantothenic acid (Vit. B5)	0.1–0.2
Magnesium	370–400	Pyridoxine (Vit. B6)	0.8
Iron	100–170	Biotin (Vit. B7)	0.01–0.055
Zinc	3–7	Folic acid (Vit. B9)	0.01–0.071
Copper	1.2	Cyanocobalamin (Vit. B12)	0.32–0.36
Manganese	3–5	Β-carotene (Provitamin A)	177–580
Selenium	0.09–0.72	Tocopherol (Vit. E)	12.5–100

**Table 5:** Examples of food products enriched with spirulina.

Product	Spirulina Addition	Benefits Received	References
Ayran	0.25%, 0.5%, 1%	Increased protein content, improved growth of probiotic bacteria	[58]
Biscuits	5%, 10%, 15%, 20%	Increase in protein content, sensory acceptability	[62]
	1%, 2.5%, 4%	At 4% addition: increase in protein and amino acid content, improvement of hardness and brittleness, colour parameters, texture, taste, and aroma	[15]
	1%, 3%, 6%	Increase in phenolic compounds and antioxidant activity, sensory acceptability, decrease in oxidative changes during storage	[59]
Bread	1.5%, 2.5%	Increase in antioxidant activity, sensory acceptability	[13]
	2%, 4%, 6%	Increase in phenolic compounds and antioxidant activity, sensory acceptability	[68]
Breadsticks	1.5%	Increase in iron and selenium content, colour stability during storage	[69]
Chicken mortadella	1%, 2%, 3%, 4%, 5%	Increase in protein, minerals, carotenoids, phenolic compounds, flavonoids, and antioxidant activity	[19]
Crostini	2%, 6%, 10%	Increase in protein content, phycocyanin, total phenolic content, antioxidant activity, sensory acceptability with lower additive	[66]
Extruded snacks	2.6%	Increase in protein, lipids, minerals, sensory acceptability	[70]
Ice cream	5%	Increase in the content of phenolic compounds, carotenoids, and antioxidant activity	[71]
Kefir	0.25%, 0.50%	Increase in phenolic compounds, antioxidant activity, promoting effect on lactobacilli and lactococci counts	[17]
	0.05%, 0.1%, 0.5%, 1%, 2%	Increase in protein, amino acids, calcium, iron, and antioxidant activity	[60]
Pasta	2.5%, 5%, 7.5%, 10%	Increase in protein content, phenolic compounds, antioxidant activity, rheological parameters, cooking quality, sensory acceptability up to 5% addition	[14]
	5%, 10%, 20%	Increase in protein content, phenolic compounds, antioxidant activity	[61]
Ricotta cheese	0%, 0.25%, 0.5%, 0.75%, 1.0%	Increase in protein, fat, ash, fibre, carbohydrates, minerals, phenolic compounds, β-carotene, and antioxidant activity; improved texture, colour, and microstructure; sensory acceptability	[72]
Wheat Crackers	2%, 6%	Increase in protein, fibre, phenolic compounds, antioxidant activity, and sensory acceptability	[73]
White chocolate	0.5%, 1%, 2%, 4%	Increase in protein, amino acids, lipids, minerals, sensory acceptability	[74]
Vegan basil pesto	1%, 2%	Increase in protein content, selected minerals, polyphenols, flavonoids, antioxidant activity, sensory acceptability	[18]
Yoghurt	0.25%, 0.5%, 0.75%, 1%	Increase in protein, fibre, iron, calcium, magnesium, carotenoids, chlorophyll, phycocyanin, antioxidant activity, better water holding capacity; sensory acceptability at 0.25% and 0.5% addition	[16]

## Data Availability

The data may be shared upon valid request.
